# Antiviral agents against COVID-19: structure-based design of specific peptidomimetic inhibitors of SARS-CoV-2 main protease

**DOI:** 10.1039/d0ra08304f

**Published:** 2020-11-04

**Authors:** Vladimir Frecer, Stanislav Miertus

**Affiliations:** Department of Physical Chemistry of Drugs, Faculty of Pharmacy, Comenius University in Bratislava Bratislava SK-83232 Slovakia frecer@fpharm.uniba.sk; International Centre for Applied Research and Sustainable Technology (ICARST) Bratislava SK-84104 Slovakia; Department of Biotechnology, Faculty of Natural Sciences, University of Ss. Cyril and Methodius Trnava SK-91701 Slovakia

## Abstract

Despite the intense development of vaccines and antiviral therapeutics, no specific treatment of coronavirus disease 2019 (COVID-19), caused by the new severe acute respiratory syndrome coronavirus 2 (SARS-CoV-2), is currently available. Recently, X-ray crystallographic structures of a validated pharmacological target of SARS-CoV-2, the main protease (M^pro^ also called 3CL^pro^) in complex with peptide-like irreversible inhibitors have been published. We have carried out computer-aided structure-based design and optimization of peptidomimetic irreversible α-ketoamide M^pro^ inhibitors and their analogues using MM, MD and QM/MM methodology, with the goal to propose lead compounds with improved binding affinity to SARS-CoV-2 M^pro^, enhanced specificity for pathogenic coronaviruses, decreased peptidic character, and favourable drug-like properties. The best inhibitor candidates designed in this work show largely improved interaction energies towards the M^pro^ and enhanced specificity due to 6 additional hydrogen bonds to the active site residues. The presented results on new SARS-CoV-2 M^pro^ inhibitors are expected to stimulate further research towards the development of specific anti-COVID-19 drugs.

## Abstract 2

Structure-based design of SARS-CoV-2 main protease inhibitors identified hydantoin, benzothiazine and cresol moieties as promising residues of new peptidomimetic inhibitors.

## Graphical abstract

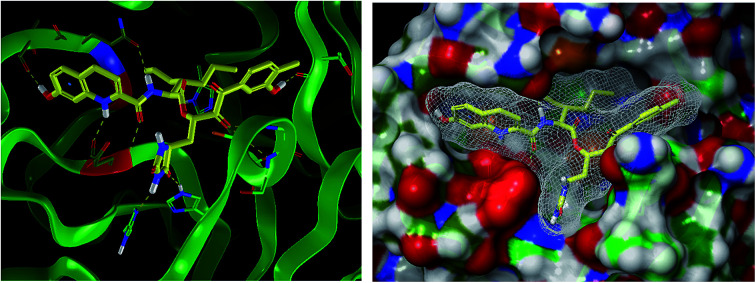

## Introduction

**Fig. 1:**
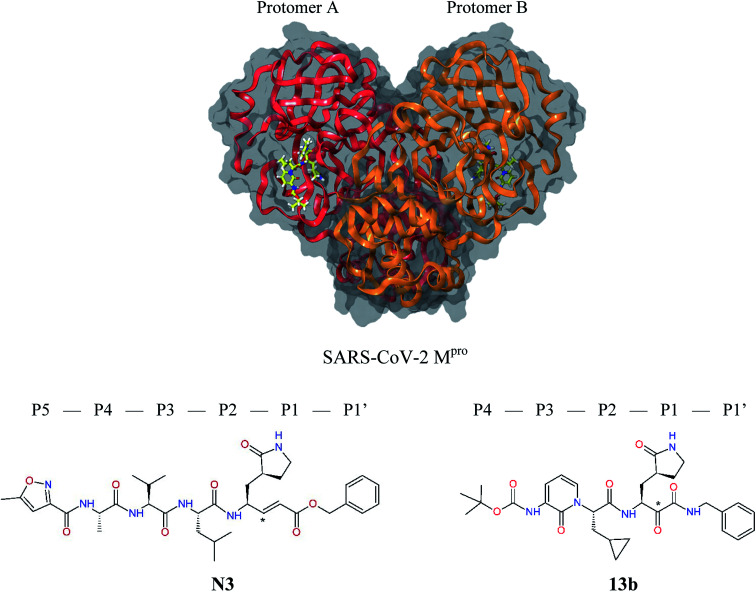
Above: ribbon representation of 3D-structure of SARS-CoV-2 M^pro^ homodimer (PDB ID 6Y2F)^12^ with inhibitor 13b (stick model, H – white, C – yellow, N – blue, O – red) bound at the active sites of the protomers A and B. Partially transparent molecular surface is shown in grey colour. Below: chemical structures of peptidomimetic covalent inhibitors N3 and 13b^12,14^ and corresponding standard notation of protease substrate residues. Stars (*) indicate the sites of nucleophilic attack of anionic sulphur of cysteine of the catalytic dyad His41 – Cys145 of the cysteine protease on the *trans*-α,β-unsaturated benzyl ester of the Michael acceptor N3^14^ or the α-ketoamide group of 13b,^12^ leading to thiohemiketal linkage formation with the Cys145.^9^

The outbreak of the new severe acute respiratory syndrome coronavirus 2 (SARS-CoV-2) belonging to the b-lineage of the betacoronaviridae family, which causes severe viral pneumonia in humans known as coronavirus disease 2019 (COVID-19), commenced in Wuhan, China in December 2019 and spread widely in 2020.^1,2^ Although intense research and development of vaccines and antiviral therapeutics is ongoing worldwide, at present only one intravenous broad-spectrum antiviral medication has been approved for treatment of COVID-19.^3^ SARS-CoV-2 is a positive-sense single-stranded enveloped RNA virus containing an RNA sequence of approx. 30 thousand bases. This unusually large viral genome codes for 4 structural proteins: spike, envelope and membrane proteins creating the viral envelope, and nucleocapsid protein holding the RNA genome, and 16 non-structural proteins (nsp1–nsp16) forming the replication–transcription complex of SARS-CoV-2.^4,5^ These proteins are expressed in the form of two polypeptides: pp1ab and pp1a, which are then processed by virally encoded chymotrypsin-like protease (called main protease M^pro^ or also 3CL^pro^), and papain-like protease (PL^pro^). The 33.8 kDa cysteine protease M^pro^ (EC 3.4.22.69) encoded in the nsp5 is a key viral enzyme essential for the viral life cycle of coronaviruses. The M^pro^ digests the larger polyprotein pp1ab (∼790 kDa) at 11 or more conserved sites with recognition sequences P3[Val, hydrophob., cationic] − P2[Leu, hydrophob., aromat.] − P1[Gln, His] + P1′[Ser, Ala, Gly] starting with an autocatalytic cleavage, to produce the functional nsps.^6–8^ In absence of closely related homologues in humans or proteases sharing similar cleavage site specificity, the M^pro^ forms an attractive pharmacological target for antiviral drug discovery.^5,9^ Recently, X-ray crystallographic structures of the M^pro^ of SARS-CoV-2 in complex with peptide-like irreversible inhibitors – Michael acceptor N3^10,11^ and α-ketoamide 13b,^12^ were resolved (PDB entries 6LU7 and 6Y2F),^12–14^Fig. 1. The N3 and 13b are potent covalent inhibitors of the SARS-CoV (2003) M^pro^ that act through a two-step irreversible inactivation mechanism. The inhibitor first associates with the M^pro^ to form enzyme-inhibitor complex (E⋯I) with an equilibrium binding constant. Then, a stable covalent bond is formed between the inhibitors and M^pro^*via* nucleophilic attack of the catalytic cysteine upon the vinyl group of N3 or α-ketoamide group of 13b (Fig. 1), characterized by high rate constant of M^pro^ inactivation resulting in a thiohemiketal formation.^10,14^ The N3 shows notable structural similarity to rupintrivir (AG-7088), a peptidomimetic antiviral drug which inhibits 3CL^pro^ proteases of rhinoviruses and is investigated also for treatment of infections caused by picornaviruses, norovirus, and coronaviruses, such as SARS.^4,15–17^

The 3D structure of SARS-CoV-2 M^pro^ forms, like the main proteases of other coronaviruses, a C_2_-symmetric dimer of two protomers A and B, each composed of three domains. Domains I and II (residues 8–184) contain six-stranded antiparallel β-barrels that adopt the chymotrypsin fold, while domain III (residues 201–306) forms α-helical structure connected to domain II by a long linking loop (residues 185–200).^5,9,10,12–14^ Active site of the M^pro^ containing Cys–His catalytic dyad, in which the Cys145 behaves as the nucleophile, while the His41 acts as the general acid/base. The substrate binding pocket is in a shallow cleft between domains I and II bordered by residues 164–168 of a long β-strand on one side and the linking loop residues 189–191 on the other side. The N-terminal part of each monomer (N-finger, residues 1–7) takes part in the M^pro^ dimerization and formation of the active site of the other monomer by interacting with Glu166, a residue important for the substrate recognition and inhibitor binding.^9,10,12,18^ The subsite S1 of the M^pro^ confers almost absolute specificity for the Gln residues of substrate. The binding site of M^pro^ is highly conserved among all coronavirus species suggesting that inhibitors targeting this site should display broad-spectrum anti-coronavirus activity.^12,14^ Therefore, the binding site of M^pro^ is likely to remain constant and less susceptible to drug-resistance conferring mutations also in the progeny strains of the SARS-CoV-2 virus of the 2019/20 season.

Since the emergence of SARS in 2003 and identification of coronavirus as the causative agent of the disease, a considerable number of peptidomimetic and small-molecule inhibitors of the M^pro^ were developed. These antiviral compounds comprise also covalent inhibitors using reactive warhead groups, which include Michael acceptors, aldehydes, epoxy ketones, electrophilic ketones such as halomethyl ketones, trifluoromethyl ketones and α-ketoamides.^9,13,18–23^ Unfortunately, due to reactivity, potential for toxicity and undesired side-effects, rapid *in vivo* metabolism and reduced oral bioavailability, the irreversible inhibitors are less likely to make efficient therapeutic agents.^9,14^ In fact, till present, there is no effective antiviral therapy for the treatment of SARS, MERS, and COVID-19 in humans.^12,24,25^ The price for lacking chemotherapeutics for coronaviruses in terms of lost human lives is too high and can be even higher in the future.

Due to the extent and death toll of the present SARS-CoV-2 pandemic and limited therapeutic options, it is rather urgent to develop improved reversible or irreversible M^pro^ inhibitors as potential antiviral agents for the treatment of COVID-19. The structure of SARS-CoV-2 M^pro^ in complex with N3,^14^13b^12^ and series of α-ketoamide inhibitors 11a–11u^23^ provided a solid ground for design and discovery of new coronavirus inhibitors. We have performed computer-assisted structure-based design and optimization of peptidomimetic α-ketoamide M^pro^ inhibitors validated by a QSAR model starting from compounds published in ref. 10, 12 and 23 and their reversible analogues with the aim to propose antiviral lead compounds with improved specificity and binding affinity to SARS-CoV-2 M^pro^, decreased peptidic character and favourable drug-like properties. The inhibitor design employed molecular mechanics (MM), conformational searching, validation by a QSAR model, molecular dynamics (MD), and was also supported by rigorous quantum chemistry method (QM/MM).

## Methods

### Receptor preparation

The 3D-structures of the SARS-CoV-2 M^pro^ in complex with covalent inhibitors N3 and 13b were obtained from recently published crystallographic data stored in the Protein Data Bank^26^ (PDB ID: 6LU7 and 6Y2F).^12,14^ In these complexes, the electrophilic warheads – unsaturated benzyl ester of the Michael-acceptor N3 and α-ketoamide of 13b (Fig. 1) were covalently linked to the catalytic residue Cys145.^10–12^ These covalent bonds were removed. Protonation and tautomeric states at physiological pH of amino acids of the M^pro^ and of inhibitors were assigned according to predicted p*K*_a_ values computed by Epik.^27,28^ All crystallographic water molecules and other crystallization additives were removed. The complexes were refined by molecular mechanics (MM) energy minimization to a minimum on the potential energy landscape by employing Polak–Ribière conjugate gradient method with convergence criterion set to energy gradient of 0.01 kJ mol^−1^ Å^−1^. During the geometry optimization an extended cut-off distance of 20 Å was used for electrostatic interactions. The OPLS3e force field, which is suitable for simulations of small molecules and proteins,^29^ together with implicit GB/SA solvent model (water),^30^ were employed throughout the minimization in MacroModel (Schrödinger, LLC.).^31^ Complexes of SARS-CoV-2 M^pro^ with other known or new inhibitors were prepared by *in situ* modification of the ligands N3 and 13b in the relaxed consensus structure of the M^pro^-N3 and M^pro^-13b complexes (6Y2F^12^ and 6LU7,^14^ rmsd = 1.79 Å for 26 active site residues, 420 atoms), by changing the side chains or backbone atoms of P3 to P1′ residues into novel molecular fragments. Initial conformations of the built-in function groups were selected according to location of energy minima on the conformational maps for rotations over rotatable bonds of the side chains, which were explored by dihedral angle coordinate scans of MacroModel,^31^ as illustrated in ref. 35–38. Total MM energy of the reference unliganded SARS-CoV-2 M^pro^ was obtained by relaxation of the proteases after removing the inhibitor by energy-minimization as the lower energy state of the two M^pro^ structures. Total energies of free inhibitors in solution was determined by conformational searching using mixed torsional/low-mode sampling method, which combines random changes in torsion angles and/or molecular position with exploring low-vibrational-frequency eigenvectors of the system that are related to ‘soft’ degrees of freedom, such as the variable torsion angles,^39^ followed by energy minimization of generated conformers.^31^

### QSAR model

To prepare a QSAR model of inhibition of the coronavirus M^pro^ by peptidomimetics we have considered a series of eleven α-ketoamides 11a–11u^23^ with consistent experimental inhibition potencies IC_50_^exp^ determined for the SARS-CoV M^pro^ (2003).^23^ The M^pro^ of SARS-CoV-2 (2019/20) and SARS-CoV (2003) share very high degree of sequence identity. Crystal structure of the (2003) SARS-CoV M^pro^-11a complex (PDB ID 5N19 (ref. 40)) was used to model the complexes of the training set inhibitors.^23^ The P2 residue side chain conformations of the peptidomimetic inhibitors were modelled by *in situ* modification of the ligand of the M^pro^-11a complex combined with a 360-degree conformational search over the torsion angles of C_α_–C_β_ and C_β_–C_γ_ bonds with an increment of 10 degrees using the coordinate scan of MacroModel.^31^ The correlation between computed relative interaction energies ΔΔ*E*_int,MM_ of the series 11a–11u^23^ and experimental inhibition potencies IC_50_^exp^ was obtained by linear regression and outliers removal. Due to limited number of the α-ketoamide inhibitors with IC_50_^exp^ data splitting of the series into training set and test set was not appropriate.

### Molecular dynamics simulations

Conformational stability of the modelled ligands and interactions at the active site of the M^pro^-inhibitor complexes were tested by molecular dynamics (MD) simulations. We have carried out 200 ns long simulations of the solvated M^pro^-inhibitor complexes in the NPT statistical ensemble (300 K, 1 bar) by using Desmond.^32^ A periodic box with 10 Å buffer containing the M^pro^-inhibitor complex was filled with approx. 11 000 TIP3P water molecules and neutralized by adding four Na^+^ ions to reach the electro-neutrality. During the simulation, OPLS3e force field, 1.5 fs integration step, and coulombic interaction cut-off of 14 Å, were used. The Nose–Hoover chains thermostat and Martyna–Tobias–Klein barostat methods were employed during the simulations.^33,34^ After initial heating and relaxation, the productive simulation trajectory was recorder and analysed for ligand-receptor interactions every 400 ps.

### Interaction energy calculations

The MM interaction energies of inhibitors to M^pro^ were calculated by the supermolecular approach (for details see the footnote of Table 1).^35–38^ In addition to MM calculations, we have computed the inhibitor binding energies by hybrid quantum mechanical/molecular mechanical (QM/MM) approach using the density functional theory by employing QSite^41,42^ and Jaguar.^43–45^ In the M^pro^-inhibitor complexes, the quantum region of the QM/MM calculation included the inhibitor and selected polar active site residues forming hydrogen bonds (HBs) or polar interactions with the ligands. The quantum region included the ligand (82 to 99 atoms) plus active site residues: Gln19sc, Thr26·, His41sc, Asn142–Gly143–Ser144–Cys145, His163sc, ·Glu166·, His172sc, Gln189–Thr190· (155 atoms of the protease, sc – side chain only, (·) – an additional bond along the backbone was included into the quantum region, total charge *q* = −1*e*). The individual residues or groups were terminated by H-caps and electrostatic interactions of atoms in the MM region neighbouring the H-caps with the quantum motif were represented by a Gaussian grid.^44^ Full geometry optimizations of the enzyme – inhibitor systems were done using DFT-M06-2X/6-311++G(d,p)//MM-OPLS-2005 level of theory using electronic embedding and analytic gradients with default convergence criteria and extended electrostatic interaction cut-off (20 Å).^44^ The embedding consists of coulombic interaction (OPLS-2005 point charges of the MM region within cut-off distance explicitly contribute to one-electron part of the QM Hamiltonian) and van der Waals interaction between the QM and MM regions (OPLS-2005 force field parameters are used for atoms of both regions).^44^ The effect of solvent was included by means of implicit solvation model as a single point calculation for the optimized geometry DFT-M06-2X/6-311++G(d,p)//MM-OPLS-2005-PBF (water). The meta-GGA global exchange-correlation density functional M06-2X with Hartree–Fock exchange is widely employed in computational chemistry for calculating energy-related quantities, and was recommended for describing thermochemistry, kinetics and non-covalent interactions by its developers.^46,47^ The flexible split-valence triple-zeta basis set with additional polarization and diffuse functions on all atoms 6-311++G(d,p)^48^ was previously found to lead to satisfactory predicted molecular geometries.^49^ In the QM/MM calculations the OPLS-2005 force field was employed,^41,42,50^ since until recently it was the only force field available in QSite with parameterization suitable for describing the interface between quantum and classical regions.^44^ The Poisson–Boltzmann continuum solvation model (PBF)^51^ that uses finite-element method on a high-resolution grid was employed to mimic the effect of the physiological aqueous environment on the molecular structure and ligand-receptor binding.^45^ The disadvantage of the QM/MM calculations using larger basis sets and extensive quantum regions, such as inhibitor bound to enzyme active site, is in rather high computational costs.††The basis set of the QM region of M^pro^-13b complex consisted of 3662 basis functions (966 electrons). The computer run time for the full QM/MM geometry optimization of this complex (approx. 150 iterations) on 32 threads of Intel Xeon Silver (16 core, AVX-512 FMA), lasted approximately 12 days.

**Table 1:** Comparison of computed relative MM interaction energies of known inhibitors to the SARS-CoV-2 M^pro^ deriving from crystal structures 6Y2F and 6LU7 (ref. 12 and 14)

Inhibitor	Formula: P5–P4–P3–P2–P1–P1′	ΔΔ*E*_int,MM_^a^ [kcal mol^−1^]	*M*_w_ b [g mol^−1^]	IC_50_^exp^^c^ SARS-CoV (2003) M^pro^ [μM]	IC_50_^exp^^d^ SARS-CoV-2 (2019/20) M^pro^ [μM]
13a^e^^,^^f^	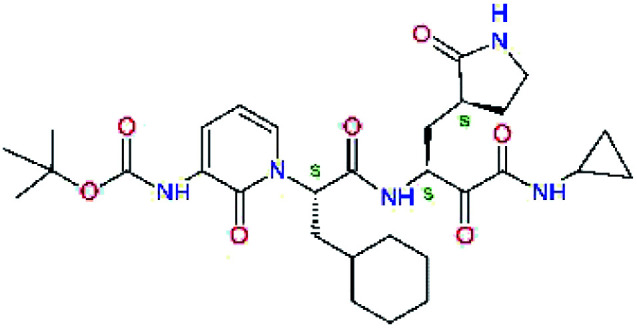	4.4	583.7	—	2.39
13b^f^	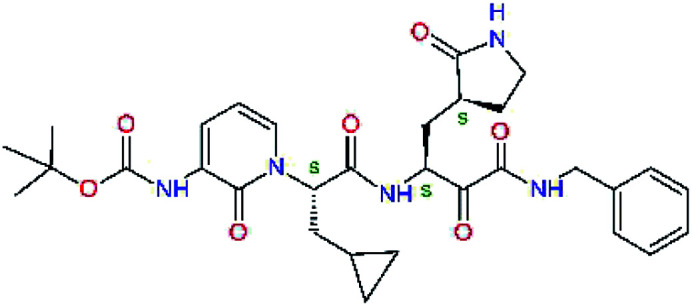	0.0^i^	591.7	0.90	0.67
N3^g^	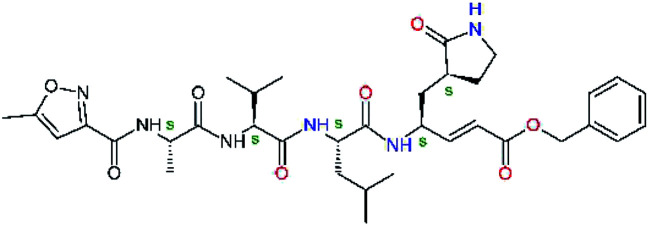	−4.1	680.8	—	—
11n^h^	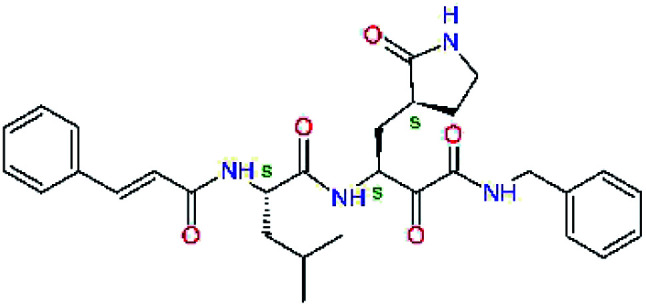	7.6	532.6	0.33	—
11r^f^^,^^h^	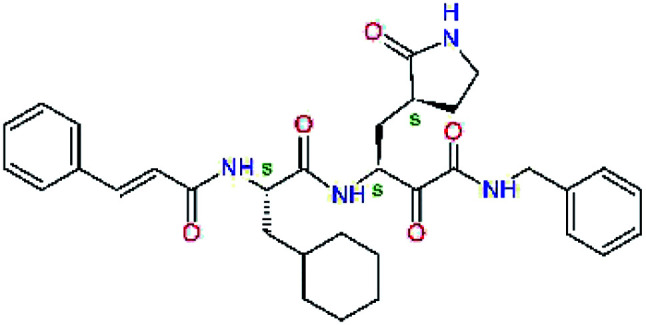	5.6	572.7	0.71	0.18

a Relative interaction energy taken with respect to the reference inhibitor 13b was calculated by molecular mechanics (MM-OPLS3e) in solution: ΔΔ*E*_int,MM_ = Δ*E*_int,MM_(I_*x*_) − Δ*E*_int,MM_(13b) = [*E*_tot,MM_{M^pro^–I_*x*_}_aq_ − *E*_tot,MM_{M^pro^}_aq_ − *E*_tot,MM_{I_*x*_}_aq_] − Δ*E*_int,MM_(13b), where *E*_tot,MM_ is total energy of solvated enzyme-inhibitor complex {M^pro^–I_*x*_}_aq_, solvated enzyme {M^pro^}_aq_, or solvated inhibitor {I_*x*_}_aq_.^35–38^ The relative interaction energy ΔΔ*E*_int,MM_ describes changes in bonding and non-bonding components of potential energy of the enzyme and inhibitor upon the enzyme-inhibitor complex formation.

b Molar mass.

c Half-maximal inhibitory concentration determined in enzyme-inhibition assay for the M^pro^ of SARS-CoV from the 2003 outbreak.^14,23^

d Half-maximal inhibitory concentration determined in enzyme-inhibition assay for the M^pro^ of SARS-CoV-2 from the 2019/20 outbreak.^12^

e Interaction energy of irreversible Michael acceptor or α-ketoamide inhibitors was computed after breaking the covalent bond of the P1 residue to the catalytic Cys145.

f Taken from ref. 12.

g Taken from ref. 14.

h Taken from ref. 23.

i The 13b was used as the reference inhibitor in all calculations of the relative interaction energy ΔΔ*E*_int,MM_.

## Results and discussion

### Inhibitor design

Recently, the Hilgenfeld laboratory described a series of peptidomimetic α-ketoamides as broad-spectrum covalent inhibitors of coronavirus and enterovirus replication^23^ and proposed some modifications directed against the main protease of the SARS-CoV-2 (2019/20).^12^ Based on their work, we decided to carry on the inhibitor optimization, explore a wider section of the chemical space of covalent and non-covalent tight-binding peptidomimetic inhibitors, and propose new potent lead compounds specific for the SARS-CoV-2 M^pro^ by means of computer-aided drug design. The optimization procedure departed from the crystal structures of SARS-CoV-2 M^pro^ in complex with the Michael-acceptor N3 (PDB ID 6LU7),^14^ and α-ketoamide 13b (PDB ID 6Y2F),^12^ which were recently made available in the Protein Data Bank.^26^ Both inhibitors N3 and 13b are bound at the active site of the M^pro^ in predominantly extended conformations,^11,12,14^ it was therefore possible to model the binding of peptidomimetic inhibitors, which share some of P1 to P3 residues with the N3 or 13b, by *in situ* modification of the M^pro^-inhibitor crystal structures.^12,14^ To determine a quantity related to the binding affinity of inhibitors to the M^pro^, we have computed relative interaction energies (ΔΔ*E*_int,MM_) of known and designed inhibitors using molecular mechanics (MM) and hybrid QM/MM approach (ΔΔ*E*_int,QM/MM_, see the Methods section). The MM interaction energy guided us in the search for more potent M^pro^ inhibitors generated by structure-based design. Table 1 gives computed ΔΔ*E*_int,MM_ of known M^pro^ inhibitors together with their experimental half-maximal inhibitory concentrations (IC_50_^exp^) determined in enzyme inhibition assays on the SARS-CoV M^pro^ (2003) and the SARS-CoV-2 M^pro^ (2019/20).^12,14,23^ All inhibitors shown in Table 1 share the γ-lactam derivative of glutamine as the P1 residue, which was found to enhance the inhibitory potency compared to the flexible glutamine side chain, most probably due to reduction of entropy loss upon binding to the M^pro^.^23,52,53^ The largest inhibitor N3 containing unsaturated benzyl ester electrophilic linkage, which occupies binding site specificity pockets S5 to S1′ and satisfies the known cleavage site preference of SARS-CoV M^pro^ for P1 to P3, displayed the strongest computed interaction energy. Unfortunately, the IC_50_^exp^ value for the N3 inhibition of SARS-CoV-2 M^pro^ is not known.^14^ On the other hand, the IC_50_^exp^ values of the α-ketoamide inhibitors 11r^23^ and 13b^12^ against the M^pro^ of SARS-CoV (2003) and SARS-CoV-2 (2019/20), are available and fall into submicromolar concentration range (Table 1).^12^ As it can be expected from the 96% sequence identity and similar 3D-structures of both viral proteases the IC_50_^exp^ of these two viral strains are rather similar.

### QSAR model of M^pro^ inhibition

**Fig. 2:**
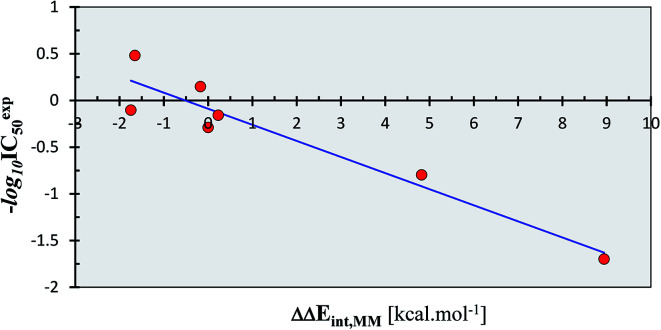
QSAR model of SARS-CoV (2003) M^pro^ inhibition by a training set of peptidomimetic α-ketoamide inhibitors^23^ (Table 2). Plot of correlation equation: −log_10_ IC_50_^exp^ = −0.1723 × ΔΔ*E*_int,MM_ − 0.0890 obtained by linear regression. Number of compounds: *n* = 11, number of removed outliers: *n*_o_ = 4, squared regression coefficient: *R*^2^ = 0.91, leave-one-out cross-validated squared regression coefficient: *R*_xv_^2^ = 0.87, statistical significance of the regression (Fisher *F*-test): *F* = 48.62, standard error: se = 0.23, level of statistical significance: *α* > 95%.

Experimental activities of the SARS-CoV-2 M^pro^ inhibition are available only for a couple of compounds (Table 1,^12,14^) and do not permit direct validation of our computational design approach by comparison of computed ΔΔ*E*_int,MM_ with the observed potencies IC_50_^exp^. However, a training set of eleven peptidomimetic α-ketoamide inhibitors 11a–11u were recently assayed for the M^pro^ of SARS-CoV (2003) inhibition.^23^ The 306 residues of M^pro^ of SARS-CoV (2003) display a 96% overall sequence identity to the M^pro^ of SARS-CoV-2 (2019/20). Moreover, for the 32 residues located within the distance of 5 Å form the bound inhibitor 13b (6Y2F),^12^ forming the core of the M^pro^ active site, the sequence identity increased to 97% (31 out of 32 residues) with a single conservative residue replacement Ser46Ala. Thus, we have validated the design approach and computed M^pro^-inhibitor relative interaction energies ΔΔ*E*_int,MM_ by preparing a QSAR model of the SARS-CoV (2003) M^pro^ inhibition (PDB entry 5N19,^40^), Table 2 and Fig. 2. The structural variability of the training set is restricted to the P2 residue only, consequently, the range of observed activities of this set of inhibitors is relatively narrow (3 orders of magnitude). Nevertheless, in the absence of other consistent sets of activity data of peptidomimetic inhibitors of SASR-CoV-2 M^pro^ in the literature, this series can be used for the validation of the computational approach.

**Table 2:** Computed relative interaction energies and observed half-maximal inhibition concentrations of a training set of peptidomimetic α-ketoamide inhibitors of M^pro^ of SARS-CoV (2003)^23^^,^^a^

Inhibitor	Formula: P3–P2–P1–P1′	ΔΔ*E*_int,MM_^*a*^ [kcal mol^−1^]	IC_50_^exp*c*^ SARS-CoV M^pro^ [μM]	−log_10_ IC_50_^exp^
11a	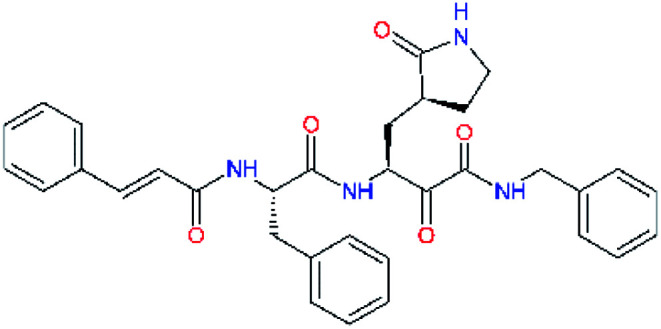	0.0	1.95 ± 0.24	−0.290
11f	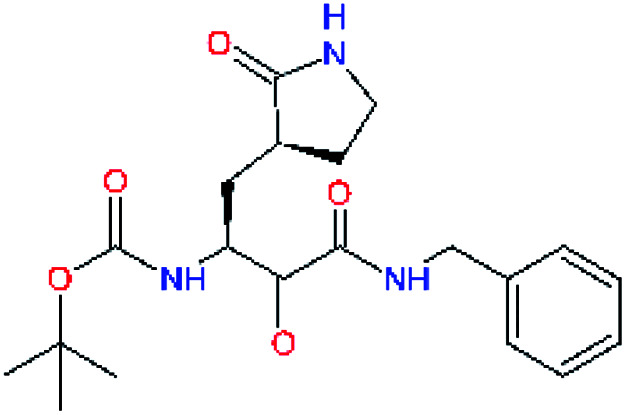	8.9	>50	−1.699
11m	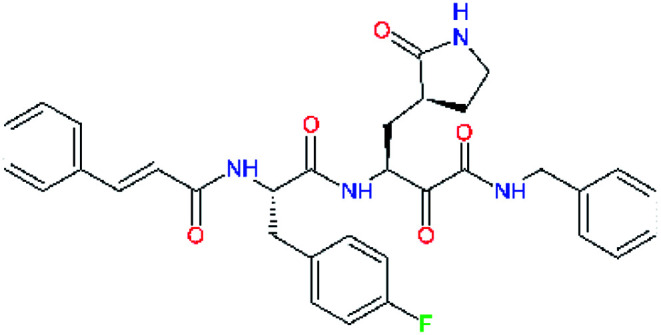	−1.9	>50	−1.699
11n	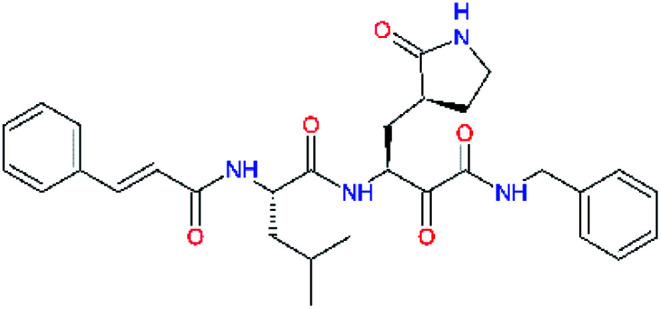	−1.7	0.33 ± 0.04	0.481
11o	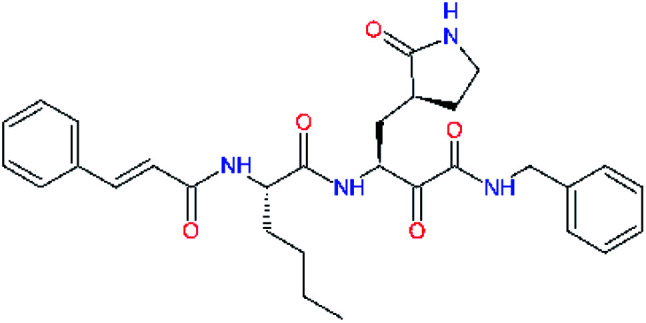	−0.3	8.50 ± 3.71	−0.929
11p	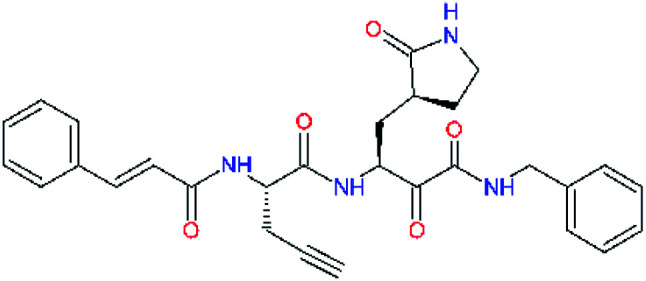	−1.1	10.68 ± 7.34	−1.028
11q	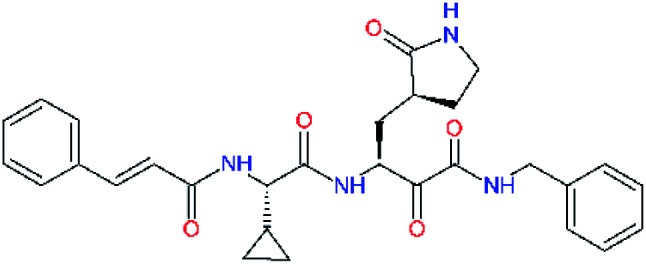	4.8	6.27 ± 2.87	−0.797
11r	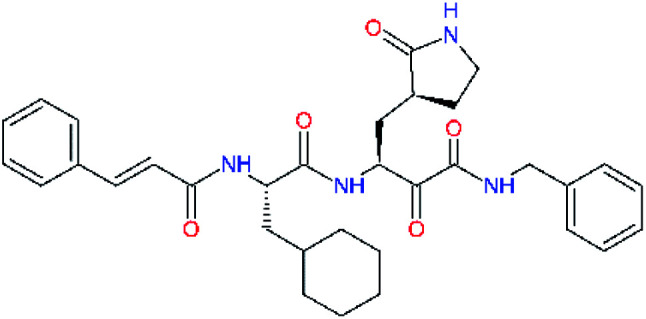	−0.2	0.71 ± 0.36	0.149
11s	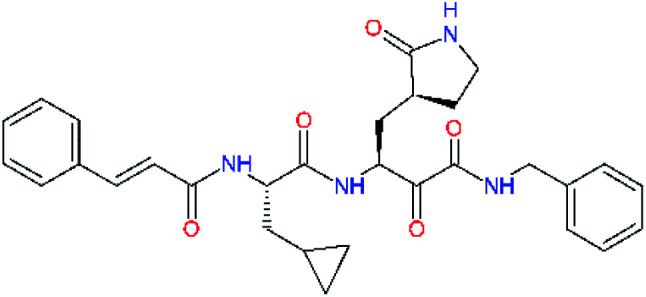	6.3	0.24 ± 0.08	0.619
11t	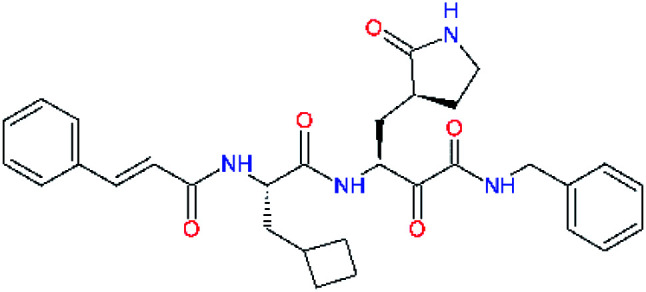	0.2	1.44 ± 0.40	−0.158
11u	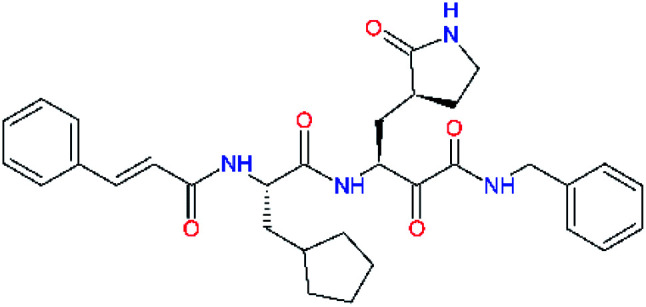	−1.7	1.27 ± 0.34	−0.104

a ^*a*,*c*^See the footnote of Table 1. Reference inhibitor 11a. Outliers are marked by purple colour.

As we can see from the statistical parameters of the correlation (Fig. 2), the computed MM relative interaction energy in solution ΔΔ*E*_int,MM_ can fairly well reproduce the observed differences in the inhibitory activities of the peptidomimetic α-ketoamides towards the M^pro^. This correlation explained about 91% of the IC_50_^exp^ data variation and underlined the role of the enthalpic contribution to the binding affinity of the peptidomimetic inhibitors (entropic effects of the ligand binding were not considered in this correlation). Therefore, we believe that the structural model and computational procedure can be applied to predict binding of similar peptidomimetics also to the M^pro^ of SARS-CoV-2 (2019/20).

### Structure-based inhibitor optimization

We have modelled the potent α-ketoamide inhibitor 11n^23^ at the binding site of the SARS-CoV-2 M^pro^,^12,14^ and optimized the P1 residue that fills the specificity pocket S1 of the protease. The S1 subsite is formed by the side chains of residues Phe140, Asn142, Glu166, His163, and His172 of protomer A and in part also by the side chain of Ser1 of the other protomer B. In addition, main chain atoms of Phe140 and Leu141 also contribute to the S1 subsite formation. This polar pocket offers the potential for more than 2 hydrogen bonds (HB) formed by the glutamine side chain. Therefore, we have explored the replacement of Glu side chain by γ-lactam, unsaturated γ-lactams, and hydantoin moieties, Table 3. When comparing the ΔΔ*E*_int,MM_ values (representing here the approximate binding affinity of the ligands to M^pro^) of the inhibitor candidates C1 to C4, the hydantoin heterocyclic moiety improved the predicted binding by more than 5 kcal mol^−1^. The reason for this enhancement is that in the relaxed structure of the M^pro^-C4 complex the P1 residue penetrates deeper into the S1 pocket compared to N3 or 13b and forms four HBs with the main-chain of Phe140 and side chains of Asn142, His163, and His172. The main-chain carbonyl group of the P1 residue makes 3 HBs with the backbone NH groups of Gly143, Ser144, and Cys145, which create the canonical “oxyanion hole” of the M^pro^ cysteine protease.^12,14^ Therefore, we retained the *glutamine hydantoin* residue as the best building block in the P1 position of all following inhibitor candidates.

**Table 3:** Optimization of P1 residue of new candidates for the SARS-CoV-2 M^pro^ α-ketoamide inhibitors^a^

Compound	Formula: P3–P2–P1–P1′	ΔΔ*E*_int,MM_^*a*^ [kcal mol^−1^]	*M*_w_^*b*^ [g mol^−1^]
C1	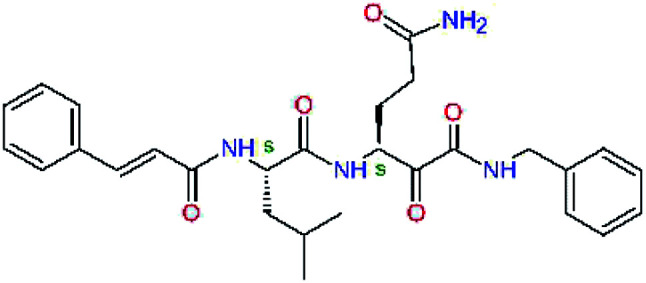	6.6	506.6
11n	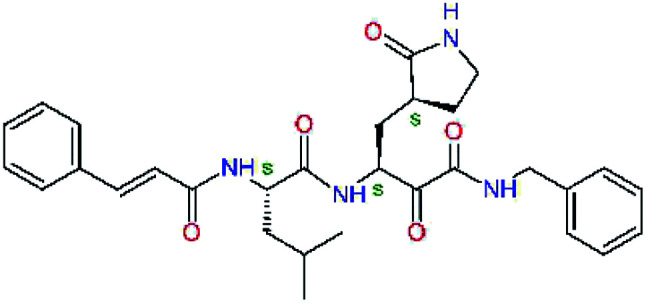	7.6	532.6
C2	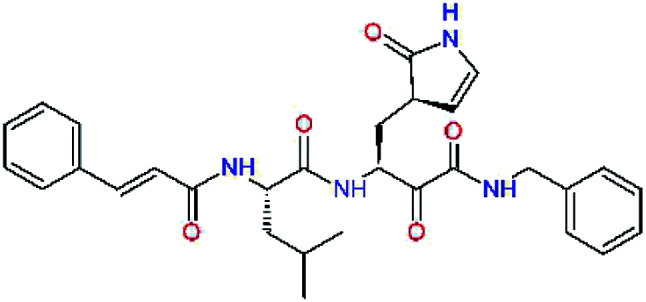	3.5	530.6
C3	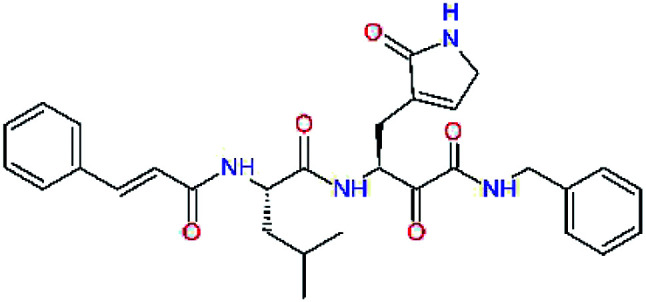	5.2	530.6
C4	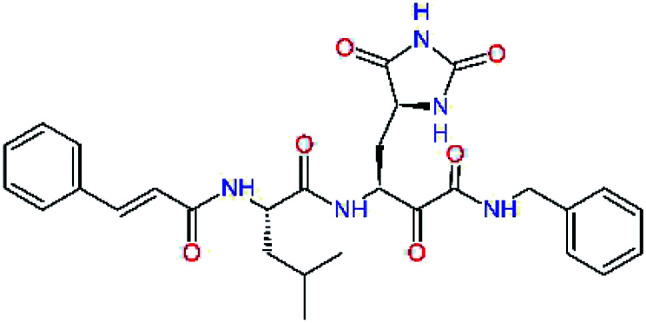	1.3	547.6

a ^*a*,*b*^See the footnote of Table 1.

**Fig. 3:**
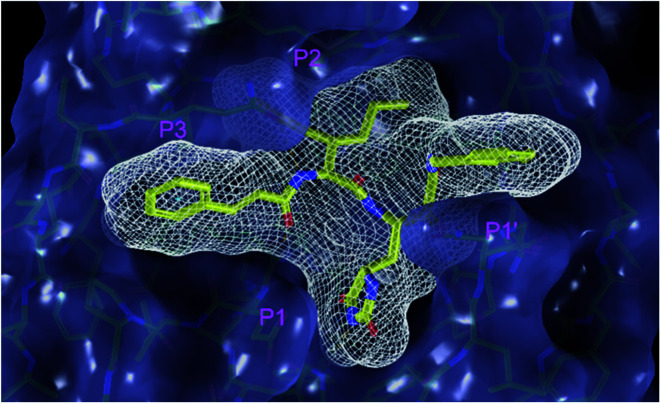
Partially transparent molecular surface of the SARS-CoV-2 M^pro^ binding site with bound inhibitor candidate C9 in stick representation (yellow – carbon, blue – nitrogen, red – oxygen, hydrogen atoms are not shown) and enclosed by a ligand surface (white mesh). The branched and bulky side chain of the P2 residue of C9 is harboured by the S2 pocket lined with residues His41, Met49, Tyr54, Met165 and Asp187 and also by S1′ pocket formed by residues Leu27, His41, Val42, and Cys145. The *N*-benzylformamide group in P1′ position of C9 partially sticks out of the S1′ into the solvent.

The substrate selectivity of the S2 pocket of the active site of coronaviruses M^pro^ for leucine was determined previously.^6,7^ Recently, the substrate recognition and cleavage site preference of SARS-CoV-2 M^pro^ were probed by a library of fluorogenic substrates, with glutamine in the P1 position, containing natural and a large panel of unnatural amino acids.^8^ Rut *et al.*^8^ concluded that the most preferred amino acid at the P2 position of SARS-CoV-2 M^pro^ is leucine. In addition, the S2 pocket can accommodate also other larger hydrophobic residues, such as 2-Abz, Phe(4-NO_2_), 3-Abz, β-Ala, Dht, hLeu, Met, and Ile (see table S1 of ref. 8 for the chemical structures of these unnatural a.u.). However, the substrate library of Rut *et al.*^8^ did not contain any unusual amino acids with bulkier and branched aliphatic side chains, as such chemicals are commercially not available. We have tested the preference of the S2 subsite of the SARS-CoV-2 M^pro^ for further branched or cyclic aliphatic side chains larger than that of leucine. The reason for selecting bulkier P2 residues was based on the 3D structure of inhibitor-M^pro^ complexes, which indicated that larger and branched side chains could be accommodated in the deeper S2 pocket and could extend also into the neighbouring larger hydrophobic S1′ subsite thus anchoring the inhibitor in the substrate binding pocket. The computed the ΔΔ*E*_int,MM_ values of inhibitor candidates C4–C10 (Table 4) suggest that besides leucine and isoleucine the P2 residue can almost equally well be composed of larger branched side chains, *e.g.* such as 4-isoheptane (C9), that is housed both by the S2 and S1′ subsites, Table 4 and Fig. 3. We have selected the C9 as the inhibitor with optimal P2 residue and retained it throughout optimization of the remaining residues of new inhibitors. We may assume that the larger and branched structure of the side chain of P2 residue could enhance the specificity for the SARS-CoV-2 M^pro^ over main proteases of other coronaviruses and enteroviruses which universally prefer Leu at the P2 position of their substrates.

**Table 4:** Optimization of P2 residue of new candidates for the SARS-CoV-2 M^pro^ α-ketoamide inhibitors^a^

Compound	Formula: P3–P2–P1–P1′	ΔΔ*E*_int,MM_^*a*^ [kcal mol^−1^]	*M*_w_^*b*^ [g mol^−1^]
C4	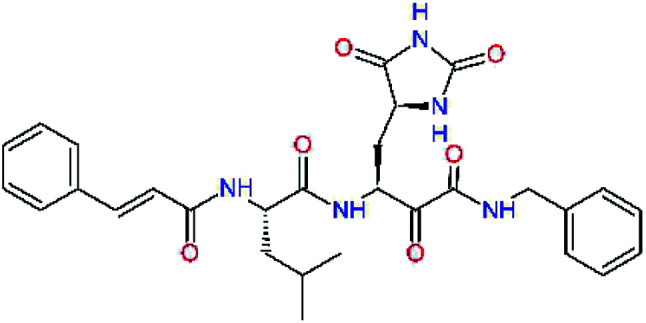	1.3	547.6
C5	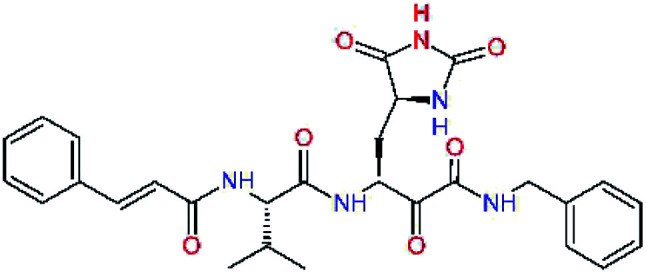	1.8	533.6
C6	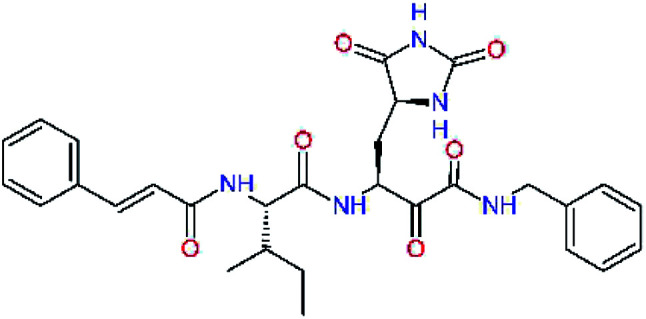	0.5	547.6
C7	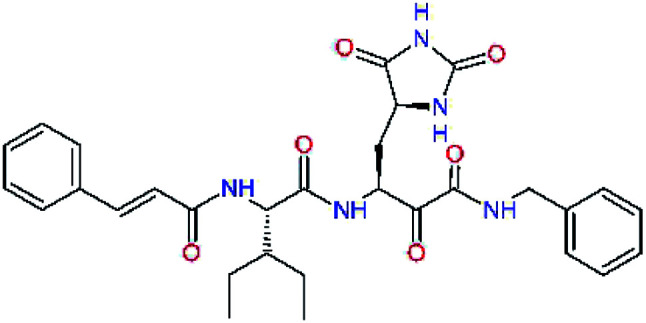	3.5	561.6
C8	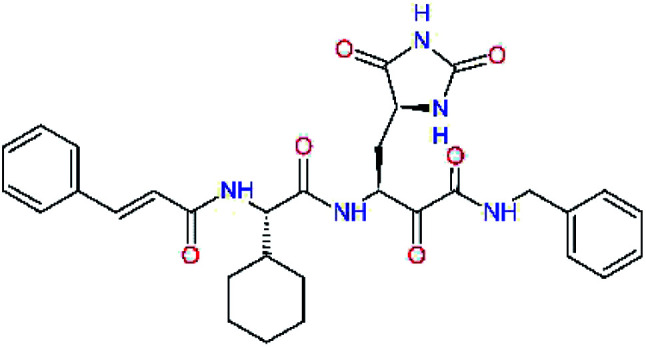	3.7	573.6
C9	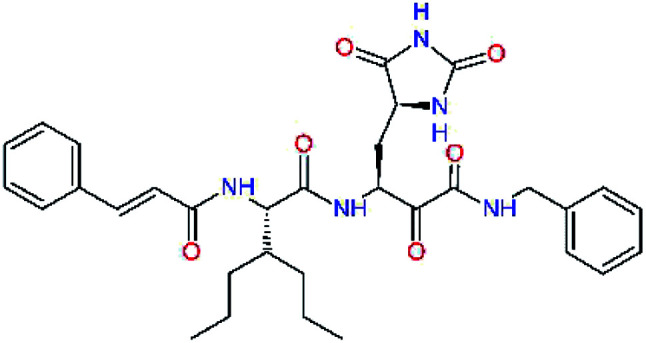	0.7	589.7
C10	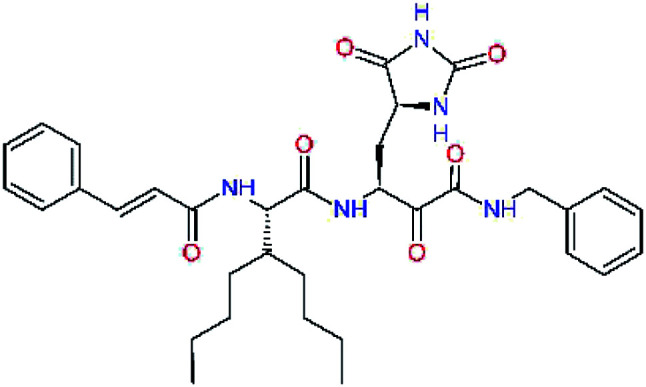	1.4	617.7

a ^*a*,*b*^See the footnote of Table 1.

The S3 subsite of the M^pro^ is solvent-exposed, which suggests that this site can tolerate a wider range of functional groups.^12,14^ To diminish unfavourable entropic effects associated with the ligand binding we decided to reduce the linker length and flexibility of the flanking P3 residue. In addition, we have extended the solvent exposed surface area of the P3 moiety by introducing rigid condensed aromatic systems that contribute to the inhibitor binding by an elevated hydrophobic effect. Moreover, we have increased the contribution of the P3 to the overall binding affinity by adding specific HB interactions to the binding site residues that occupy the S3 and S4 subsites. The S4 subsite is composed of Met165, Leu167, Pro168, Gln182, and Gln189, which can enter polar as well as nonpolar interactions with the ligand. Thus, we have introduced a hydroxyl group making a proton-donor HB to the backbone carbonyl of Thr190 (C11, Table 5). Next, we have considered introducing a larger heterocyclic moiety into the P3 position to decrease flexibility and peptidic character of the P3 residue and rise the stability of the inhibitor structure. The 1,4-dihydroquinoxaline at the P3 position brought an additional proton-donor HB to backbone carbonyl of Glu166 (C12). Other condensed aromatic moieties included quinoxalin-1(4*H*)-ol (C14), quinolin-4(1*H*)-one (C15), 4*H*-1,4-benzoxazine (C16), and 4*H*-1,4-benzothiazine (C17). Various heterocycles were considered for the P3 residues with the goal to gain an additional HB of the heteroatoms or function groups to the side chain of Gln189. In addition, the substituted fused ring of the P3 residue of C12 to C17 contribute to the ligand binding by a lone pair – π interaction with the nitrogen of Pro168.^54,55^ Out of the inhibitor candidates with modified P3 residue the C17 appeared as the most perspective one displaying binding affinity enhancement by more than 4 kcal mol^−1^ compared to C9 (Table 4), and increased specificity of binding by 2 additional HBs to the residues of the M^pro^ active site. Interestingly, the hydroxylamine function group of quinoxaline in C14 showed to be less favourable than the thioether group of benzothiazine in C17 probably due to its elevated hydrophobic character.

**Table 5:** Optimization of P3 residue of new candidates for the SARS-CoV-2 M^pro^ α-ketoamide inhibitors^a^

Compound	Formula: P3–P2–P1–P1′	ΔΔ*E*_int,MM_^*a*^ [kcal mol^−1^]	*M*_w_^*b*^ [g mol^−1^]
C9	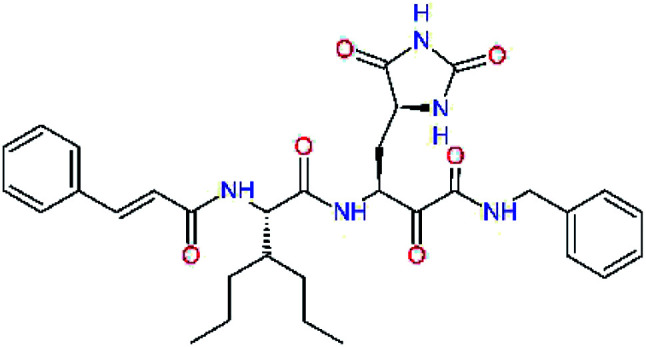	0.7	589.7
C11	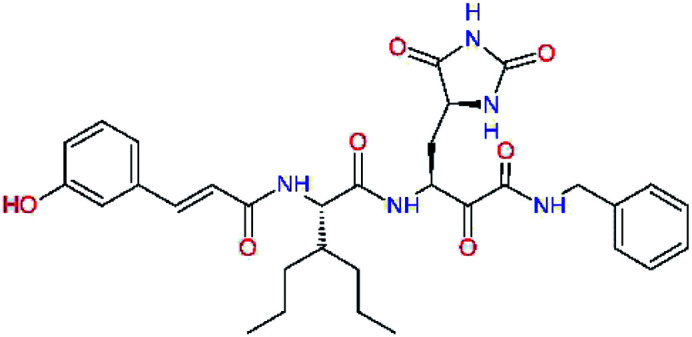	1.1	605.7
C12	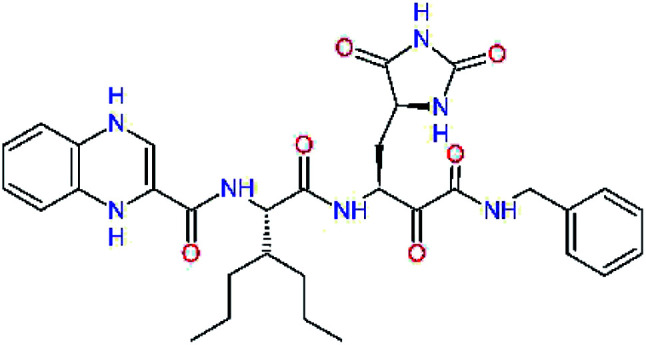	0.0	617.7
C13	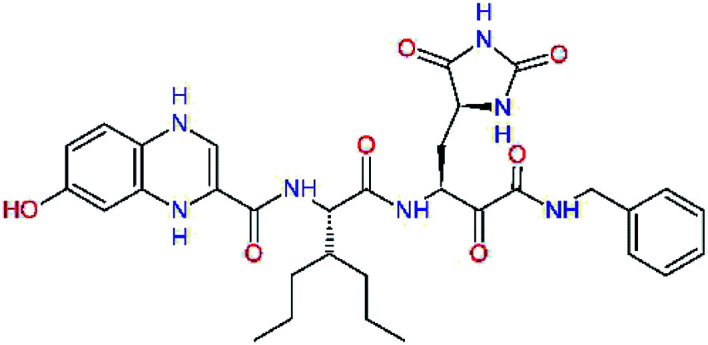	−0.9	633.7
C14	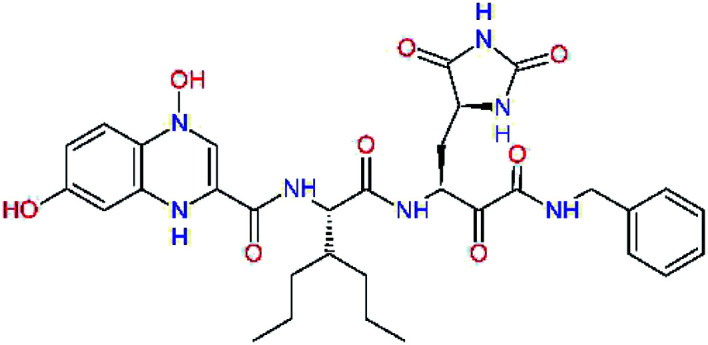	−2.8	649.7
C15	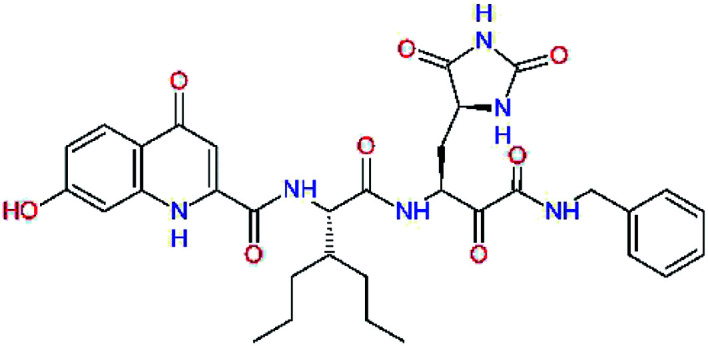	−0.5	646.7
C16	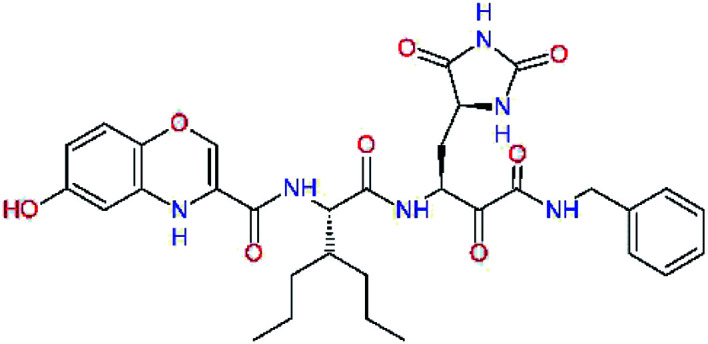	−0.7	634.7
C17	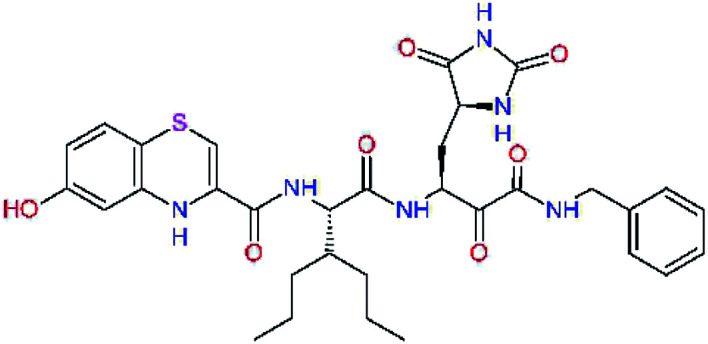	−3.6	650.7

a ^*a*,*b*^See the footnote of Table 1.

The S1′ site of SARS-CoV-2 M^pro^ (2019/20) is formed chiefly by hydrophobic residues Leu27, His41, Val42, and Cys145 and the P1′ benzyl ester portion of the inhibitor N3 is protruding out of the S1′ into the solvent.^14^ Alike the P3 residue optimization strategy, the improvement of the P1′ building block involved shortening of the linkage, introducing condensed aromatic system that could partially occupy the hydrophobic S1′ subsite, and adding new function groups able to interact with Gln19 located at the edge of the S1′ pocket (C17–C23, Table 5). Naphthalene diol of C20, which forms a proton-donor HBs to the side chain of Gln19 appeared as the most suitable replacement of the P1′ phenylmethanamine of the C17. This modification enhanced the binding affinity of C20 to the M^pro^ by over 11 kcal mol^−1^ compared to C17 (Table 6). Other benzenediol, naphthalenediol, phenol, or hydroxynaphthalene groups in C17–C23 proved to be less effective.

**Table 6:** Optimization of P1′ residue of new candidates for the SARS-CoV-2 M^pro^ α-ketoamide inhibitors^a^

Compound	Formula: P3–P2–P1–P1′	ΔΔ*E*_int,MM_^*a*^ [kcal mol^−1^]	*M*_w_^*b*^ [g mol^−1^]
C17	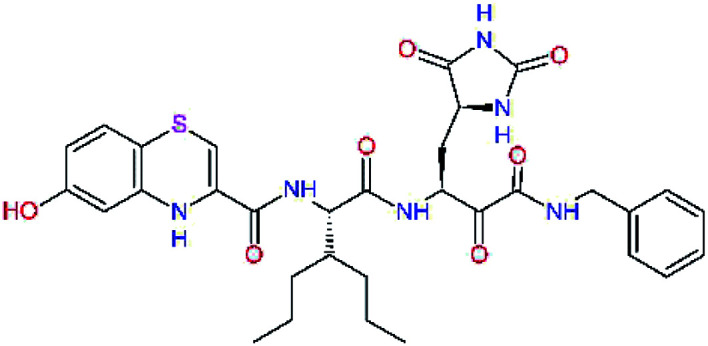	−3.6	650.7
C18	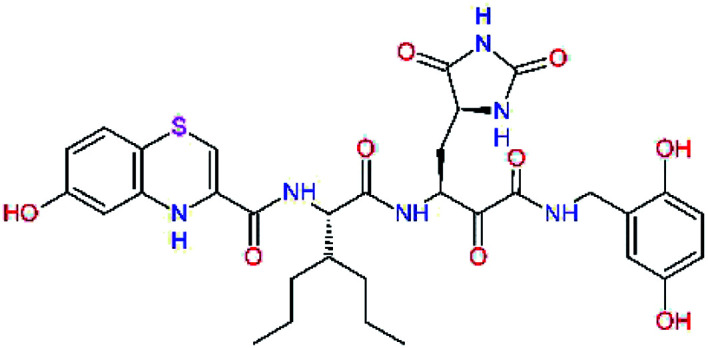	−1.3	682.7
C19	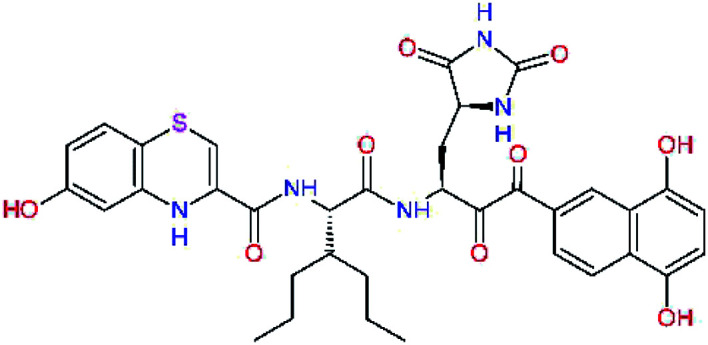	−12.2	703.8
C20	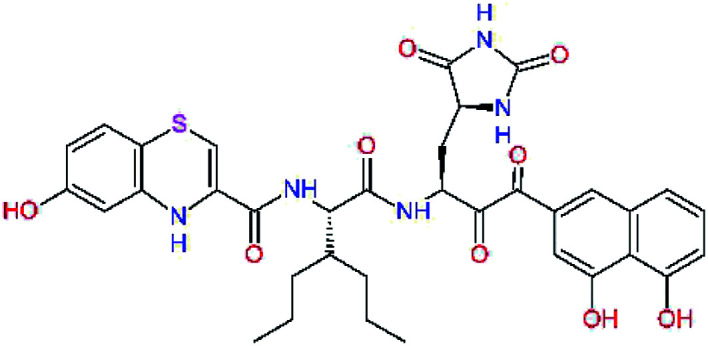	−14.7	703.8
C21	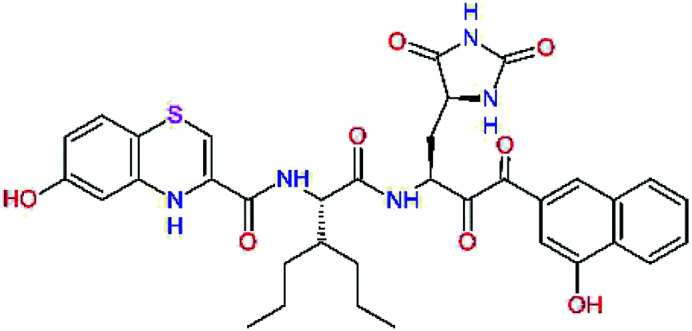	−11.4	687.8
C22	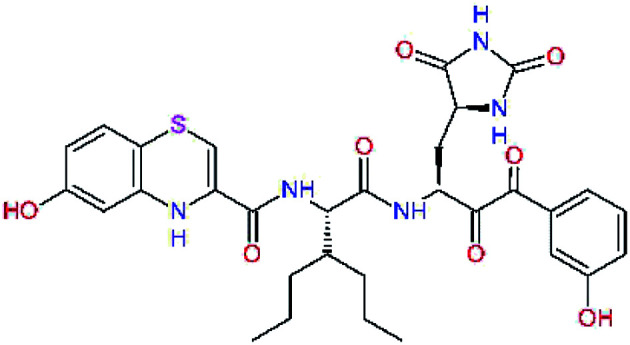	−9.1	637.7
C23	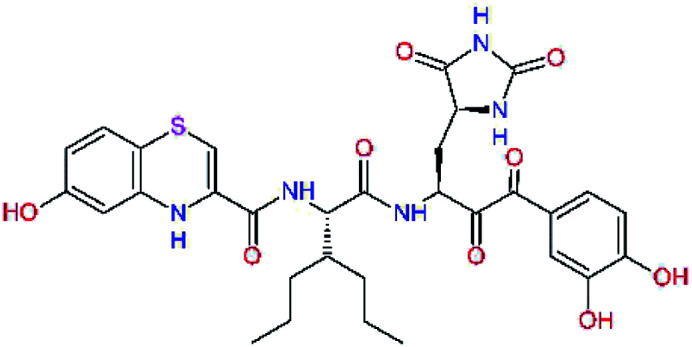	−8.5	653.7

a ^*a*,*b*^See the footnote of Table 1.

In the Table 7 we present computed binding affinities of inhibitor candidates C20–C28, which contain structural modifications leading to increased proteolytic stability of the peptidomimetic inhibitors. We have adopted replacements of the α-ketoamide linkage and peptide bonds by isosteres non-cleavable by proteases to reduce reactivity, potential toxicity, side effects and instability of the covalent inhibitors (C24–C26). In addition, we have tested introduction of a rigid cyclic molecular core that could stabilize the peptidomimetics in their bound conformation (C27, C28). The non-covalent inhibitor candidate C25 with diacetyl group replaced by the ester linkage appears as a plausible alternative peptidomimetic inhibitor candidate of the SARS-CoV-2 M^pro^.

**Table 7:** Replacement of peptide bonds and cyclization of the SARS-CoV-2 M^pro^ α-ketoamide inhibitor candidates^a^

Compound	Formula: P3–P2–P1–P1′	ΔΔE_int,MM_^*a*^ [kcal mol^−1^]	*M*_w_^*b*^ [g mol^−1^]
C20	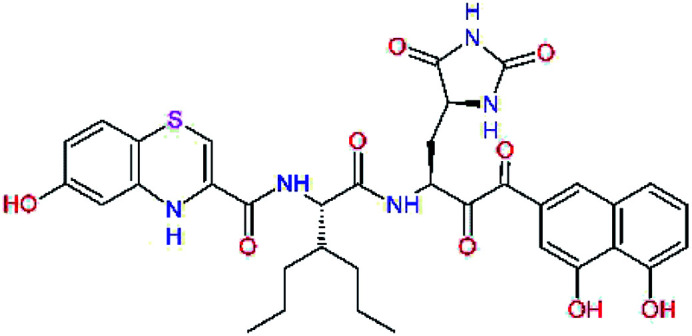	−14.7	703.8
C24	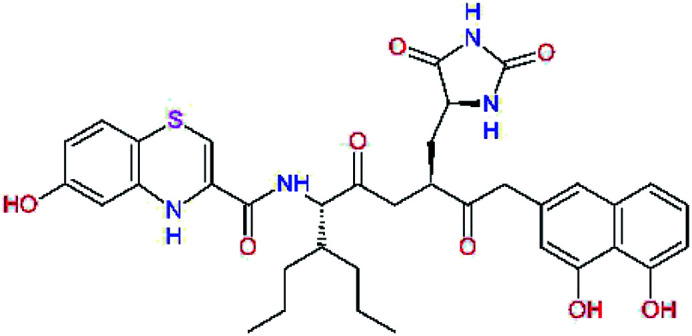	−10.9	688.8
C25	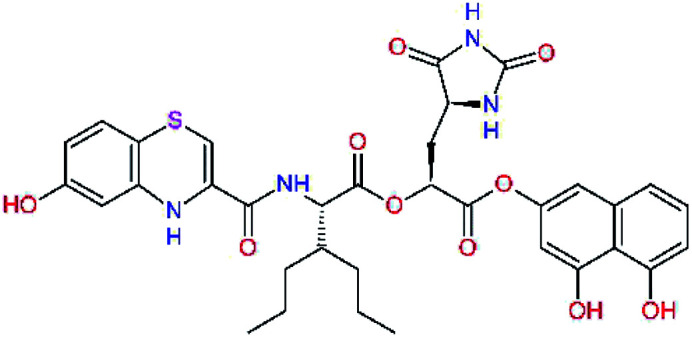	−14.5	692.7
C26	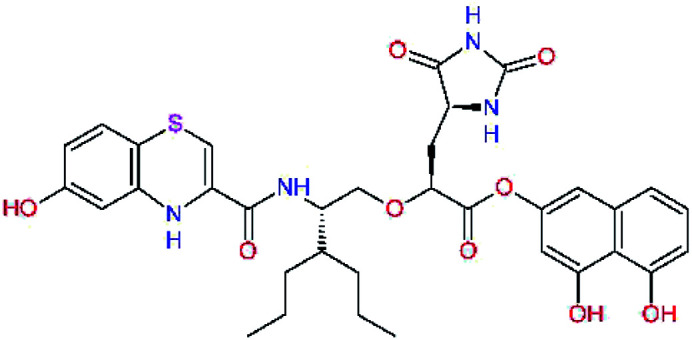	−6.6	678.7
C27	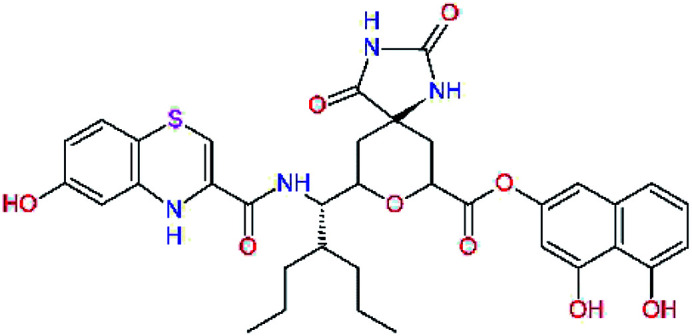	−5.9	690.8
C28	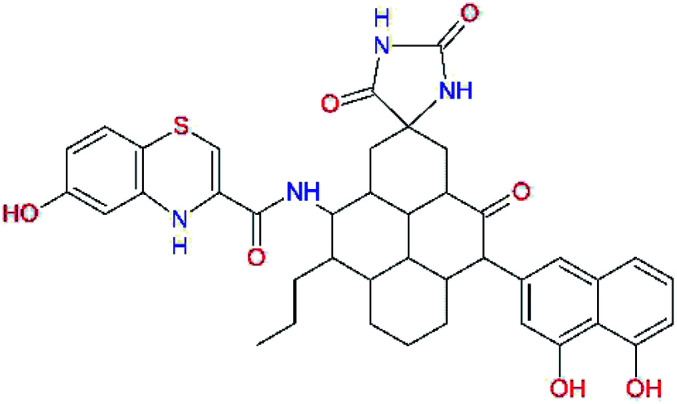	−6.3	722.8

a ^*a*,*b*^See the footnote of Table 1.

**Fig. 4:**
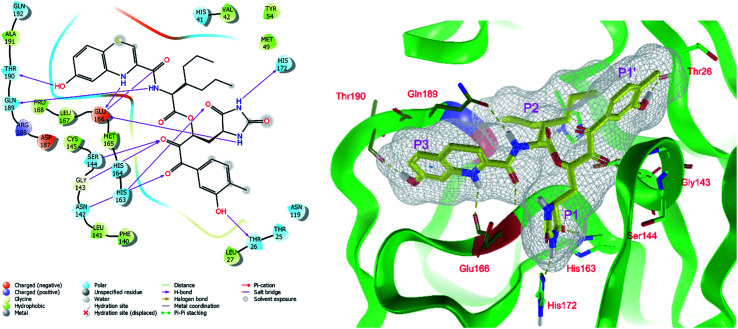
Left: 2D-interactions scheme of inhibitor candidate C33 at the SARS-CoV-2 M^pro^ binding site optimized by MM. Right: 3D structure of inhibitor C33 bound to M^pro^ in tube representation (yellow – carbon, blue – nitrogen, red – oxygen, beige – sulphur, hydrogens are not displayed). Hydrogen bonds are shown as yellow dashed lines. The protein ribbon is coloured by residue charge (blue – cationic, green – neutral, red – anionic).

In the last optimization step, we have evaluated whether additional combinations of the flanking P3 and P1′ residues and peptide and ester linkage replacements could lead to smaller, stable and more potent inhibitors. Table 8 shows that the best inhibitor structures with the lowest computed relative interaction energy to the SARS-CoV-2 M^pro^ are the butanedione linked candidates C31 and C33 with phenol or *o*-cresol at the P1′ position, modest molecular mass, and 9 HBs to residues: Thr26, Gly143, Ser144, His163, Glu166, His172, Gln189, and Thr190, Fig. 4. The 2 substituents on the phenyl ring of P1′ residue in C33 and C34 form HBs and van der Waals contacts with the main-chain and side chain of Thr26. These interactions in combination with the effect of solvent contribute favourably to the ligand binding. It is interesting to notice that replacement of ester bridge to the P1′ residue with the butanedione linkage enhanced the affinity of the peptidomimetic inhibitors towards the M^pro^. Also, analogue C34 containing the ester linkage of the P1′ residue represents a promising lead compound, which displays predicted interaction energy to the SARS-CoV-2 M^pro^ better than the submicromolar inhibitors 13b and 11r (Table 8).^12^

**Table 8:** Cooperativity between P3, P2, P1 and P1′ residues and downsizing of optimized SARS-CoV-2 M^pro^ α-ketoamide and ester inhibitor candidates^a^

Compound	Formula: P3–P2–P1–P1′	ΔΔ*E*_int,MM_^*a*^ [kcal mol^−1^]	*M*_w_^*b*^ [g mol^−1^]
C25	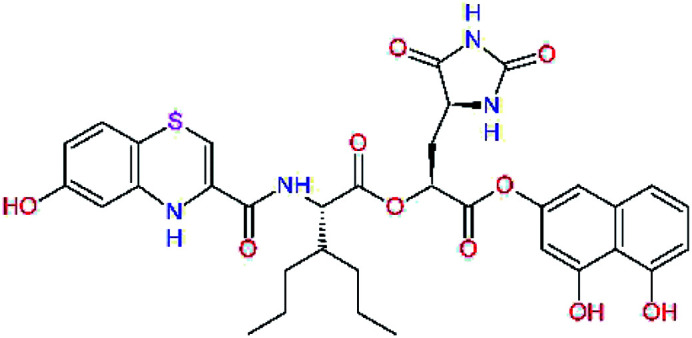	−14.5	692.7
C29	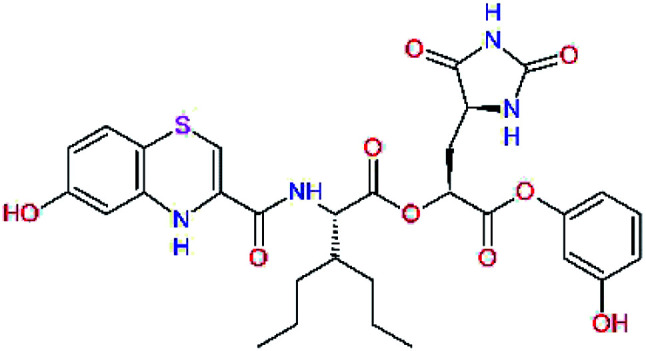	−14.4	626.7
C30	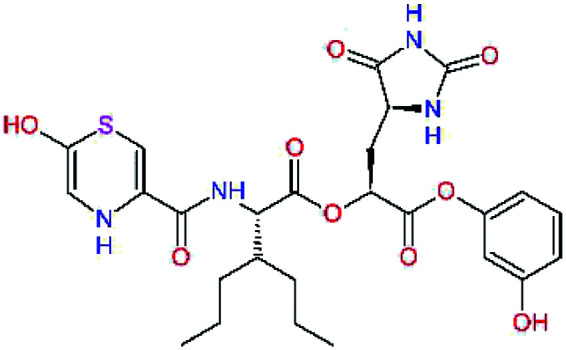	−11.2	560.6
C31	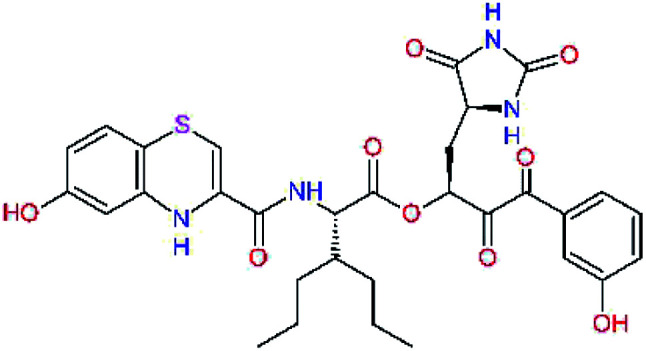	−16.3	638.7
C32	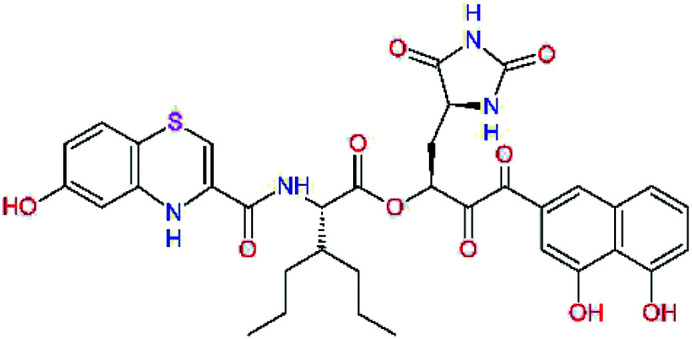	−15.8	704.7
C33	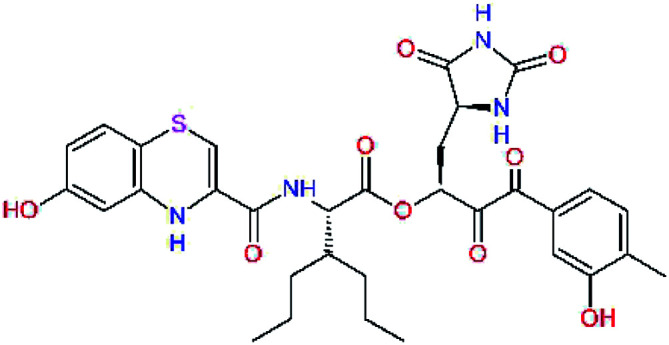	−16.7	652.7
C34	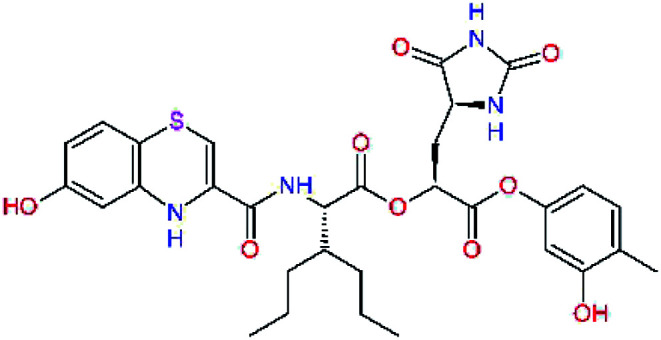	−16.8	640.7

a ^*a*,*b*^See the footnote of Table 1.

### MD simulations

**Fig. 5:**
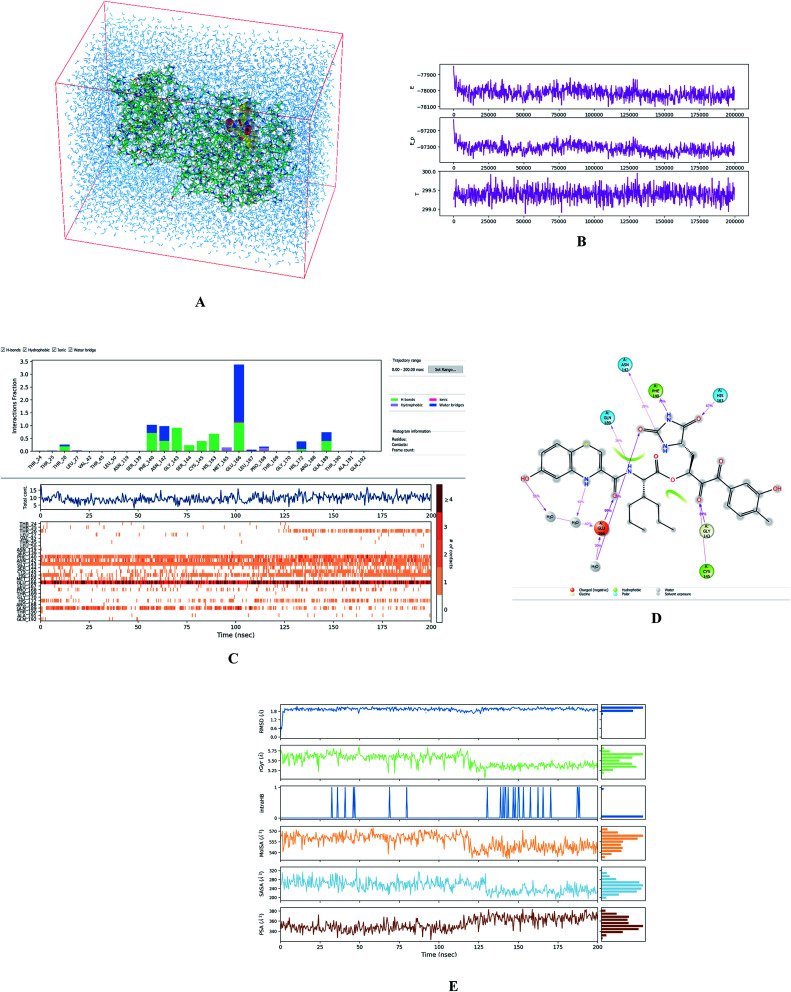
(A) Periodic box with solvated M^pro^-C33 complex. (B) Top: plot of total energy of the system during the 200 ns simulation in Desmond^32^ (〈*E*〉 = −78 018 ± 32 kcal mol^−1^), middle: potential energy (〈*E*_p_〉 = −97 311 ± 28 kcal mol^−1^), bottom: temperature (〈*T*〉 = 299.4 ± 0.2 K). (C) Analysis of enzyme-inhibitor interactions. Top: contribution of individual active site residues to inhibitor binding (HB – green, ionic interactions – magenta, hydrophobic – purple, water bridges – blue); middle: number of favourable contacts between the M^pro^ and C33, bottom: time-evolution of the interactions between inhibitor and individual active site residues. (D) 2D representation of the most populated attractive interactions between C33 and individual active site residues of SARS-CoV-2 M^pro^ occurring at least in 1/3 of the 500 analysed frames. (E) Evolution of properties of the bound inhibitor during the simulations. Top to bottom: root mean square deviation from the initial structure (RMSD), radius of gyration (rGyr), number of intramolecular hydrogen bonds (intraHB), molecular surface area (MolSA), solvent-accessible surface area (SASA), and polar surface area (PSA).

To evaluate the stability of selected modelled M^pro^-inhibitor complexes and conformational flexibility of the bound inhibitors we have carried out 200 ns MD simulations (see the Methods section) using Desmond software.^32^Fig. 5 illustrates the simulated system and evolutions of active site interactions within the complexes and ligand properties during the simulation.

The assessment showed that complexes of the most stable proposed inhibitor candidates C31–C34 preserve the binding mode as well as the ligand conformation during the MD simulation. The binding analysis also indicated that the main contribution to the inhibitor binding originates from HB and polar interactions of the P3 residue with the Glu166, which is essential for the structure of M^pro^ dimer and its catalytic function.^9,10,12,18^ The other significant contribution to the inhibitor binding comes from the novel *glutamine hydantoin* residue P1 that maintains the HBs with Phe140, His163, and His172 residues. Also, other substitutions guided by the M^pro^ structure, such as introduction of polar hydroxyl groups to the aromatic rings of flanking P3 and P1′ residues, which bind through HBs to the Thr26, Thr190, and Gln192, and water molecules, were preserved during most of the MD simulation time and stabilized the M^pro^-C33 complex (Fig. 5C and D). The results of MD simulations confirmed the validity of the binding mode of peptidomimetic inhibitors predicted from modelling of the M^pro^-inhibitor complexes and MM calculations.

### QM/MM calculations

**Fig. 6:**
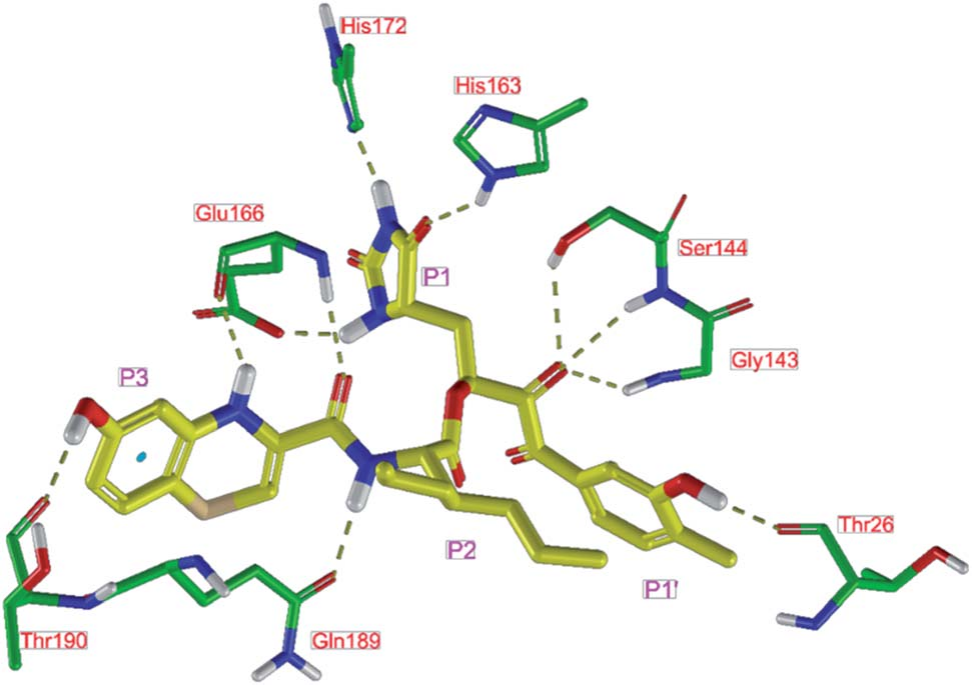
Detailed view of HB interactions of inhibitor candidate C33 bound to the active site of SARS-CoV-2 M^pro^ obtained by QM/MM geometry optimization of the enzyme inhibitor complex (in tube representation, yellow – carbon, blue – nitrogen, red – oxygen, beige – sulphur, nonpolar hydrogens are not displayed). Eleven HBs of C33 to eight M^pro^ active site residues are shown as beige dashed lines.

In the crystal structure of SARS-CoV-2 M^pro^-13b^12^ the residues P1 and P1′ of the inhibitor make polar contacts with amino acid side chains forming the S1 and S1′ subsites. Moreover, the 13b and N3 form at least 4 HBs with the main chain of the active site residues, which helps to lock the inhibitor inside the substrate binding pocket.^12^ To evaluate the binding affinities of designed inhibitors towards the M^pro^ with a higher accuracy, including also the effects of polarization, charge transfer, lone pair – aromatic interactions, and solvent polarization, we have used a more rigorous QM/MM approach at the DFT-M06-2X/6-311++G(d,p)//MM-OPLS-2005-PBF (water) level of theory (see the Methods section) for a limited number of known inhibitors and new inhibitor candidates. Table 9 gives a more accurate estimate of the enzyme – inhibitor interaction energies ΔΔ*E*_int,QM/MM_ for 3 reference inhibitors 13b, 11n and 11r,^12,23^ and 3 designed inhibitor candidates C31, C33 and C34. Both the α-ketoamide covalent inhibitors C31 and C33 as well as the ester analogue C34 show considerably better predicted interaction energies to M^pro^ then the submicromolar α-ketoamides 13b, 11n, and 11r, Table 9. The QM/MM calculations confirmed the trend in ΔΔ*E*_int,MM_ obtained by simpler MM calculations shown above (Tables 3–8). The quantum mechanical description of the enzyme-inhibitor interactions within the active site clearly favoured the more polar new molecules C31, C33 and C34 over the known inhibitors. The specificity of these lead compounds towards the SARS-CoV-2 M^pro^ is highly increased. Compared to the submicromolar inhibitor 13b, the leads C31, C33, and C34 form 6 additional HBs to the binding site residues of the M^pro^. The novel hydantoin moiety, which occupies the S1 subsite, contributes alone by 3 HBs to His163, Glu166, and His172 side chains towards the inhibitor binding affinity and specificity (Fig. 6). The compound 13b inhibits the recombinant SARS-CoV-2 M^pro^ with the IC_50_^exp^ of 0.67 μM (ref. 12) and 11r with IC_50_^exp^ = 0.18 μM, Table 9.^12,23^ The predicted significantly enhanced binding affinities of C31, C33, and C34 compared to the known inhibitors 13b, 11n, and 11r suggest that these analogues could represent new promising lead compounds worthwhile of further development.

**Table 9:** Comparison of relative enzyme-inhibitor interaction energies of known and designed α-ketoamide and ester M^pro^ inhibitors computed by the QM/MM method

Inhibitor	Formula: P3–P2–P1–P1′	ΔΔ*E*_int,QM/MM_^a^ [kcal mol^−1^]	*M*_w_ b [g mol^−1^]	*Q*_C*_ c [è]	IC_50_^exp^^d^ SARS-CoV-2 (2019/20) M^pro^ [μM]
13b^e^^,^^f^	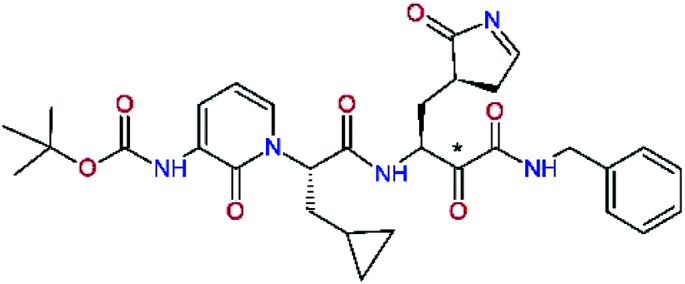	0.0^g^	591.7	0.133	0.67
11n^h^	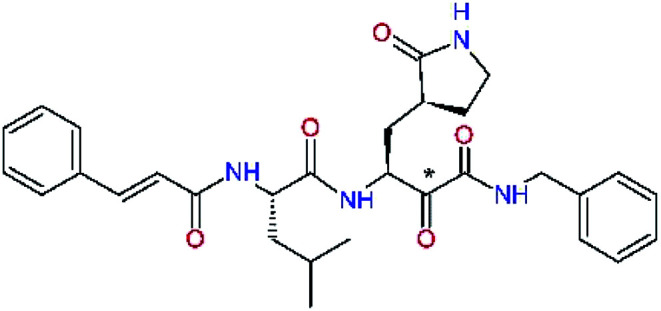	1.6	532.6	0.183	—
11r^h^	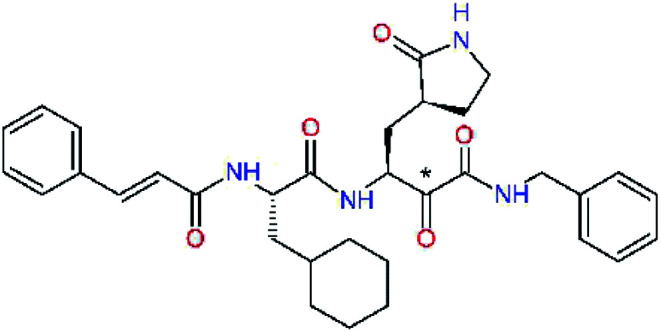	4.8	572.7	0.183	0.18
C31	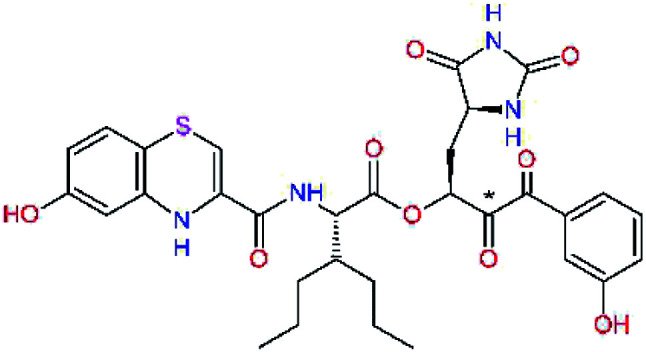	−18.6	638.7	0.143	—
C33	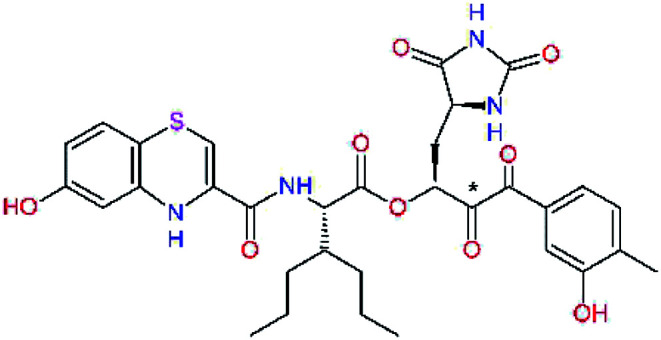	−17.4	652.7	0.150	—
C34	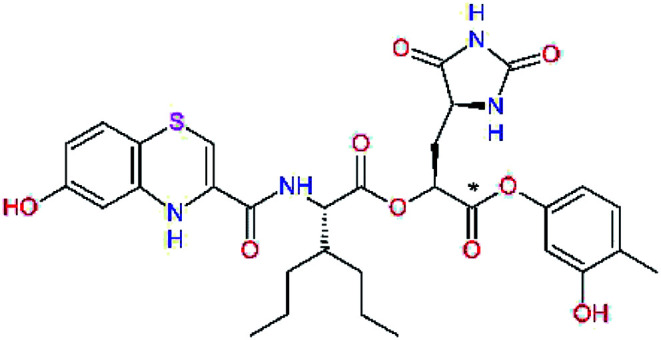	−15.2	640.7	0.315	—

a Relative enzyme-inhibitor interaction energies taken with respect to the reference inhibitor 13b were calculated by the hybrid QM/MM method DFT-M06-2X/6-311++G(d,p)//MM-OPLS-2005-PBF (water) in solution: ΔΔ*E*_int,QM/MM_ = Δ*E*_int,QM/MM_(I_*x*_) − Δ*E*_int,QM/MM_(13b) = [*E*_tot,QM/MM_{M^pro^–I_*x*_}_aq_ − *E*_tot,QM/MM_{M^pro^}_aq_ − *E*_tot,QM/MM_{I_*x*_}_aq_] − Δ*E*_int,QM/MM_(13b), where *E*_tot,QM/MM_ is total QM/MM energy of solvated enzyme-inhibitor complex {M^pro^–I_*x*_}_aq_, solvated enzyme {M^pro^}_aq_, or solvated inhibitor {I_*x*_}_aq_.

b Molar mass.

c Net Mulliken atomic charge,^56^ obtained by molecular orbital analysis, on the carbon of P1 residue (indicated by *) undergoing the nucleophilic attack of the sulphur S_γ_ of catalytic Cys145 residue during peptide bond cleavage by the M^pro^, was calculated by the DFT method in vacuum for bound inhibitor in the M^pro^–I_*x*_ complex.

d Experimental half-maximal inhibitory concentrations (IC_50_^exp^) of SARS-CoV-2 (2019/20) M^pro^ inhibition were taken from ref. 12. The experimental inhibitory activities are available only for inhibitors 13b and 11r.

e The interaction energy of the irreversible inhibitors (13b, 11n and 11r) was computed after breaking the covalent bond of their P1 residue to the catalytic Cys145.

f Taken from ref. 12.

g Reference value.

h Taken from ref. 23.

The net atomic charge (*Q*_C*_) of the electrophilic carbon atom of the carbonyl group (indicated by * in Table 9),^56^ targeted during the nucleophilic attack by the S_γ_ of the catalytic Cys145, demonstrates the susceptibility of the ligands to start formation of the covalent thiohemiketal linkage to the Cys145.^9^ As we can see from Table 9, the net charge *Q*_C*_ of inhibitor candidates C31 and C33 is comparable to that in the α-ketoamide inhibitors 13b, 11n, and 11r, which indicates similar reactivity towards nucleophiles and equivalent potential for undesired side-effects. On the other hand, the ester analogue C34 with *Q*_C*_ of 0.315*e* may represent a stronger electrophile than the reference α-ketoamide inhibitors.

### ADME properties

Many lead compounds fail at an advanced stage of pharmaceutical development due to adverse pharmacokinetic profiles.^57^ It is therefore essential to incorporate ADME properties prediction already into the lead prioritization. Therefore, we have calculated a set of 24 ADME-related descriptors of known M^pro^ inhibitors as well as the 3 best designed inhibitor candidates by the QikProp software.^58^Table 10 lists 9 selected descriptors, which were computed by the methods of Jorgensen.^59–61^ The overall drug-likeness parameter (#stars), which describes the compliance of ADME properties with the requirements for drug-like molecules, characterizes in a simple manner the pharmacokinetic profile of inhibitor candidates and can serve as a secondary compound selection criterion. The new inhibitor candidates listed in Table 10, C31, C33, and C34 display acceptable ADME properties. Therefore, these molecules can be recommended for further pharmaceutical development.

**Table 10:** Selected ADME-related properties of inhibitors of M^pro^ of SARS-CoV-2 predicted with help of QikProp^58^

Inhibitor	vRoF^a^	log *P*_o/w_^b^	log *S*_wat_^c^	log *K*_HSA_^d^	*P*_caco_ e	#metab^f^	HOA^g^	log HERG^h^	#stars^i^
13b^j^	2	2.5	−3.8	−0.4	78	6	50	−4.2	0
11n^k^	1	3.2	−3.3	−0.1	56	4	64	−3.0	0
11r^k^	1	3.9	−3.9	0.2	50	4	67	−3.1	0
C31	3	2.8	−3.5	−0.1	68	9	37	−4.8	5
C33	3	3.0	−3.7	0.1	68	9	38	−4.5	4
C34	3	3.2	−4.0	0.2	104	9	43	−4.5	1

a Number of violations of Lipinski's rule of five^62^ and drug-like character of compounds. The rule requires that: *M*_w_ < 500 Da, log *P*_o/w_ < 5, #HB_don_ ≤ 5, #HB_acc_ ≤ 10. Optimum range of values: vRoF ≤ 4.

b Logarithm of predicted octanol/water partition coefficient. Optimum range of values: −2.0 to +6.5.

c Logarithm of predicted aqueous solubility (S in [mol dm^−3^]) gives the concentration of the solute in a saturated solution that is in equilibrium with the crystalline solid. Optimum range of values: −6.5 to +0.5.

d Logarithm of predicted binding constant to human serum albumin. Optimum range of values: −1.5 to +1.5.

e Predicted apparent gut/blood barrier permeability by passive transport in [nm s^−1^] using the Caco-2 cells model. Optimum range of values: *P*_caco_ < 25 nm s^−1^ is poor, *P*_caco_ > 500 nm s^−1^ is great.

f Number of likely metabolic reactions.

g Predicted human oral absorption expressed in %. Optimum range of values: HOA > 80% is high, HOA < 25% is poor.

h Logarithm of predicted IC_50_ value for blockage of the HERG K^+^ channels. Optimum range of values: concern if log HERG <−5.

i Number of property or descriptor values that fall outside the 95% range of similar values of known drugs for 24 descriptors calculated in QikProp^58^ (the remaining 15 calculated descriptors are not shown) documents drug-like character and pharmacokinetic profile of a compound. Optimum range of values: 0–5.

j Taken from ref. 12.

k Taken from ref. 23.

## Conclusions

The SARS-CoV-2 M^pro^ was recognized as a validated pharmacological target for the design of antiviral drugs, which are urgently needed to combat the ongoing COVID-19 pandemic. Computer-aided structure-based design of reversible and irreversible inhibitors of the M^pro^ took advantage of the crystal structures of complexes of viral protease co-crystallized with peptidomimetic inhibitors N3 and 13b.^12,14^ Hydantoin, benzothiazine and cresol moieties were identified as promising design elements that can occupy S1, S3–S4, and S1′ subsites of the M^pro^ active site. Our effort resulted in the identification of new analogues C31, C33 and C34 with predicted enhanced binding affinities to M^pro^, elevated specificity, and favourable ADME-related properties. Predictions based on MM calculations and QSAR model of M^pro^ inhibition were assessed by MD simulations and qualitatively confirmed by the more comprehensive QM/MM approach.

Therefore, we encourage medicinal chemistry laboratories working in the field of drug design and development against the COVID-19 to verify our computational predictions by synthesis and enzyme inhibition assays of the proposed SARS-CoV-2 M^pro^ inhibitor candidates.

## Author contributions

The manuscript was written through contributions of all authors. All authors have given approval to the final version of the manuscript.

## Funding sources

This research was funded by the grants APVV-17-0239 and PP-COVID-20-0010 of the Slovak Research and Development Agency as well as the grant VEGA 1/0228/17 of the Granting Agency of Slovak Ministry of Education and Slovak Academy of Sciences.

## Abbreviations

3CL^pro^ or M^pro^: Main protease of SARS-CoV-2
3D: Three-dimensional
#stars: ADME-related overall drug–likeness parameter
ADME: Adsorption, distribution, metabolism, and excretion
COVID-19: Coronavirus disease 2019
DFT: Density functional theory
GB/SA: Generalized Born solvent accessible surface area
MM: Molecular mechanics
M06-2X: Minnesota class 06 hybrid exchange-correlation density functional with double amount of nonlocal exchange (2X)
MERS: Middle East respiratory syndrome
meta-GGA: Meta generalized gradient approximation functional
OPLS3e: Optimized potential for liquid simulations version 3 extended
OPLS-2005: All atom force field developed by Schrödinger for organic molecules
PBF: Poisson Boltzmann finite element method
PDB: Protein Data Bank
QM/MM: Mixed quantum mechanics/molecular mechanics approach
SARS: Severe acute respiratory syndrome

## Conflicts of interest

The authors declare no conflict of interest.

## Supplementary Material
